# Complete Genome Sequences of the Species Type Strains Sinorhizobium garamanticum LMG 24692 and Sinorhizobium numidicum LMG 27395 and CIP 109850

**DOI:** 10.1128/mra.00251-23

**Published:** 2023-05-31

**Authors:** Sabhjeet Kaur, Daniel Espinosa-Sáiz, Encarna Velázquez, Esther Menéndez, George C. diCenzo

**Affiliations:** a Department of Biology, Queen’s University, Kingston, Ontario, Canada; b Departamento de Microbiología y Genética, Instituto de Investigación en Agrobiotecnología (CIALE), Universidad de Salamanca, Salamanca, Spain; c Grupo de Interacción Planta-Microorganismo, USAL, Unidad Asociada al CSIC por el IRNASA, Salamanca, Spain; d Mediterranean Institute for Agriculture, Environment and Development (MED) and Global Change and Sustainability Institute (CHANGE), Institute for Advanced Studies and Research, University of Évora, Pólo da Mitra, Évora, Portugal; University of Arizona

## Abstract

The genus *Sinorhizobium* comprises rhizobia that fix nitrogen in symbiosis with legumes. To support taxonomic studies of this genus and of rhizobia more broadly, we report complete genome sequences and annotations for the species type strains Sinorhizobium garamanticum LMG 24692 and Sinorhizobium numidicum LMG 27395 and CIP 109850.

## ANNOUNCEMENT

The genus *Sinorhizobium* currently consists of 17 validly published species (1), all of which can enter into nitrogen-fixing symbiosis with leguminous plants. Prior to this work, the genome sequences of the species type strains for 14 *Sinorhizobium* spp. were publicly available (1–9). To support taxonomic studies of the genus *Sinorhizobium*, we report the genome sequences of type strains for two additional *Sinorhizobium* species: S. garamanticum LMG 24692 and S. numidicum LMG 27395 and CIP 109850 (10).

*S. garamanticum* LMG 24692^T^ and *S. numidicum* LMG 27395^T^ were obtained from the Belgian Coordinated Collections of Microorganisms/Laboratorium voor Microbiologie, Universiteit Gent (BCCM/LMG), in June 2010 and November 2022, respectively, while *S. numidicum* CIP 109850^T^ was obtained from the Institute Pasteur Collection in November 2022. Following rehydration, single colonies were immediately used to prepare 20% glycerol stocks for storage at −80°C until use. For DNA isolation, strains were streaked from the frozen stocks onto tryptone-yeast extract (TY) medium (11). Single colonies were then inoculated into TY broth and grown overnight at 28°C, after which DNA was extracted using Monarch genomic DNA purification kits (New England Biolabs) according to the manufacturer’s instructions. Oxford Nanopore Technologies (ONT) sequencing was performed using rapid barcoding kits (SQK-RBK004, ONT) and R9.4.1 flow cells on a MinION device. Base calling and demultiplexing were performed using Guppy version 6.3.4+cfaa134 (LMG 24692^T^) or 6.4.6+ae70e8f (LMG 27395^T^ and CIP 109850^T^), with the r941_min_sup_g507 model (ONT). Illumina sequencing was performed at SeqCenter (Pittsburgh, PA, USA) using the Illumina DNA prep kit and IDT 10-bp unique dual indexes (UDI) and sequenced on an Illumina NextSeq 2000 instrument to produce 2 × 151-bp reads. The Illumina reads were filtered using BBDuk version 38.96 (12) and trimmed using Trimmomatic version 0.39 (13) with the following parameters: LEADING:3 TRAILING:3 SLIDINGWINDOW:4:15 MINLEN:36. Sequencing statistics are provided in Table 1.

**TABLE 1:** Accession numbers and sequencing, assembly, and annotation statistics

Characteristic	Data for strain:		
	*Sinorhizobium garamanticum* LMG 24692^T^	*Sinorhizobium numidicum* LMG 27395^T^	*Sinorhizobium numidicum* CIP 109850^T^
BioProject accession no.	PRJNA935862	PRJNA935862	PRJNA935862
BioSample accession no.	SAMN33397752	SAMN33397753	SAMN33397754
GenBank Assembly accession no.	GCF_029892065.1	GCF_029891955.1	GCF_029892045.1
GenBank accession no.	CP120373, CP120374, CP120375	CP120370, CP120371, CP120372	CP120367, CP120368, CP120369
SRA accession no.			
ONT reads	SRR23576515	SRR23576513	SRR23576511
Illumina reads	SRR23576516	SRR23576514	SRR23576512
Total ONT read length (nt)^*a*^	625,602,711	344,577,201	1,137,159,815
No. of ONT reads	69,663	83,380	315,111
ONT *N*_50_ read length (nt)	15,783	7,853	6,421
Total Illumina read length (nt)	3,426,040,094	1,987,185,289	1,660,861,335
No. of Illumina paired reads	14,645,955	8,521,205	7,011,977
Illumina read length (nt)	2 × 151	2 × 151	2 × 151
Genome size (bp)	6,834,248	6,722,813	6,722,812
No. of protein coding genes	6,111	5,983	5,981
G+C content (%)	61.33	60.68	60.68
No. of replicons	3	3	3
Replicon sizes (bp)	4,218,344; 1,992,739; 623,165	3,768,519; 2,324,848; 629,446	3,768,519; 2,324,848; 629,445

a nt, nucleotides.

Draft genomes were generated from the ONT reads using Flye version 2.9-b1779 (14), and contigs less than 2,000 bp were removed. The assemblies were then polished using the ONT reads and Medaka version 1.7.2 (ONT). The assemblies were further polished with the Illumina reads, first using Polypolish version 0.5.0 (15) and then using POLCA version 4.0.9 (16); in both cases, read mapping was performed using BWA version 0.7.17-r1198-dirty (17). The fixstart option of Circlator version 1.5.5 (18) was used to reorient replicons to start at the *dnaA* gene (if found) or otherwise at a gene nearest to the middle of the replicon. Finally, the genomes were annotated using PGAP version 2022-10-03.build6384 (19). Eight threads (when multithreading was possible) and default parameters were used for all software unless otherwise specified. The assembly and annotation statistics are provided in Table 1.

**FIG 1:**
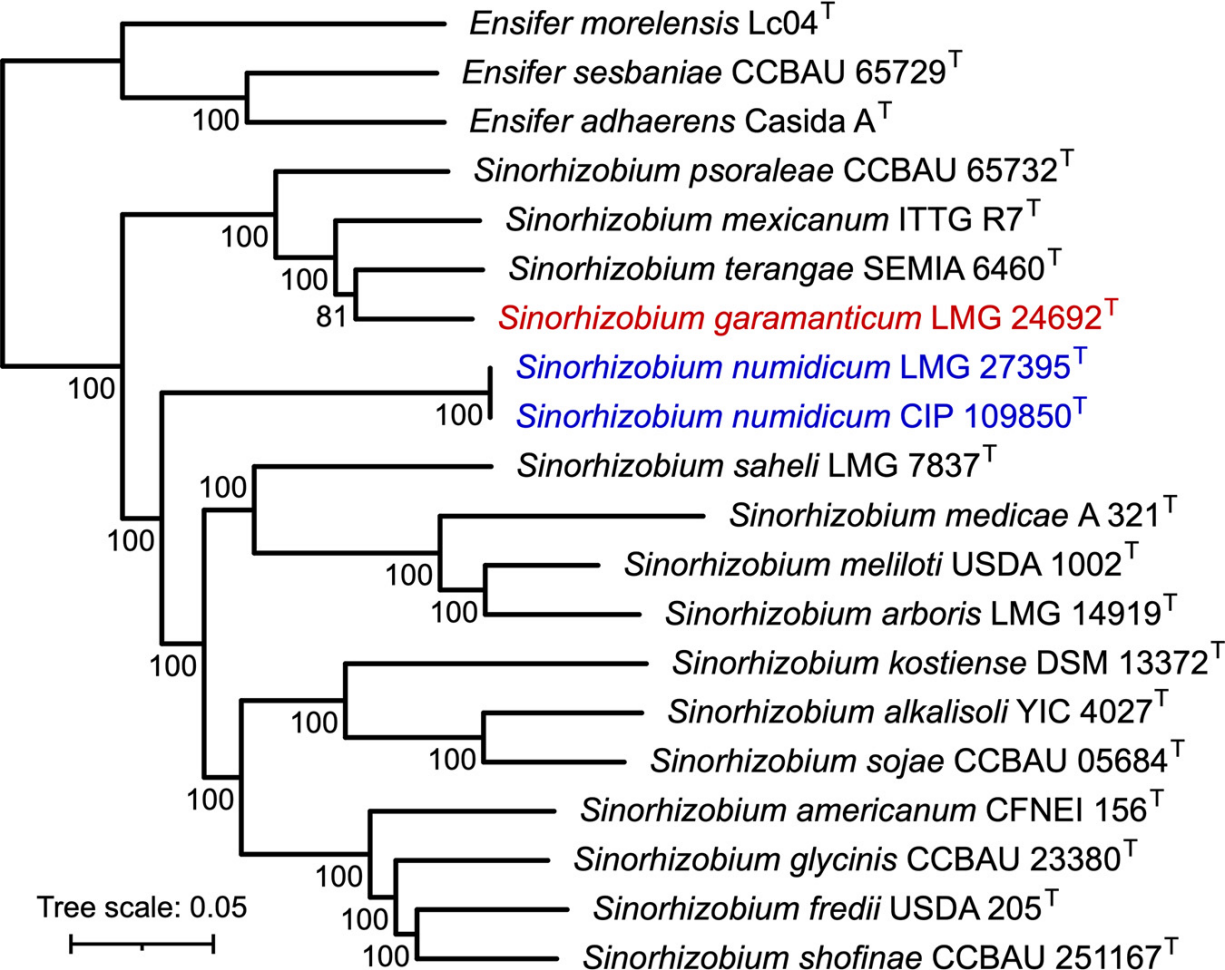
Maximum likelihood phylogeny of the genus *Sinorhizobium*. A maximum likelihood phylogeny of 16 species type strains from the genus *Sinorhizobium* was prepared from a concatenated alignment of 1,509 core genes. *S. garamanticum* is shown in red, while *S. numidicum* is shown in blue. Three *Ensifer* spp. (7, 21) were included as an outgroup. To construct the phylogeny, Roary version 1.7.8 (22) was used to identify core genes (80% identity threshold), align them individually using PRANK version 170427 (23), and concatenate the alignments. The concatenated alignment of 1,509 genes was then trimmed using trimAl version 1.4 rev22 (24) and used to construct a maximum likelihood phylogeny using IQ-TREE version 2.2.0 and the GTR+F+I+I+R5 model (25). Values at the nodes represent ultrafast jackknife support values calculated from 1,000 replicates and a subsampling proportion of 40%. The scale represents the mean number of nucleotide substitutions per site.

Pairwise average nucleotide identity comparisons against all other *Sinorhizobium* species type strains, as calculated using FastANI version 1.33 (20), confirmed *S. garamanticum* and *S. numidicum* to be distinct species, as all values were below 95%. A core genome maximum likelihood phylogeny was constructed as described previously (Fig. 1) (7). The results indicated that *S. garamanticum* forms a clade with Sinorhizobium terangae, S. mexicanum, and S. psoraleae. On the other hand, *S. numidicum* formed its own lineage, a result that differs from a previous phylogeny created using 16S rRNA gene sequences (10).

### Data availability.

The annotated genome assemblies and raw sequencing reads were deposited at NCBI GenBank and the Sequence Read Archives, respectively, and are accessible via the accession numbers listed in Table 1. Scripts to repeat the genome assembly and annotation, as well as the ANI and phylogenetic analyses, are available at GitHub (https://github.com/diCenzo-Lab/008_2023_Sinorhizobium_species_type_strains).
